# Lymphangiogenesis after nonvascularized lymph node transplantation: Lymphangiographic findings in mice and minipigs

**DOI:** 10.1371/journal.pone.0295836

**Published:** 2023-12-15

**Authors:** Kyoung Min Kim, Kun Yung Kim

**Affiliations:** 1 Departments of Pathology, Jeonbuk National University Medical School, Research Institute of Clinical Medicine of Jeonbuk National University, Biomedical Research Institute of Jeonbuk National University Hospital, Jeonju, Republic of Korea; 2 Department of Radiology, Seoul National University Bundang Hospital, Seongnam-si, Gyeonggi-do, Republic of Korea; 3 Department of Radiology, Seoul National University College of Medicine, Seoul, Republic of Korea; University Magna Graecia of Catanzaro, ITALY

## Abstract

The establishment of new connections after NVLNT (non-vascularized lymph node transplantation) is still poorly understood. The purpose of this study was to investigate lymphatic connections after NVLNT using lymphangiography. In a mice model, 40 mice were allocated to undergo NVLNT or sham surgery. On day 21 after NVLNT, the lymphatic vessels were observed on near-infrared fluorescence imaging with indocyanine green. In a minipig model, 12 minipigs underwent NVLNT. On day 14 after NVLNT, the transplanted lymph node and donor site were checked by ultrasound, and minipigs with viable transplanted LNs were allocated to lipiodol lymphangiography or MR lymphangiography groups. Transplanted LN engraftment was examined with immunohistochemical staining. After NVLNT in mice, the signal intensities in the popliteal region at 3 minutes and 5 minutes were higher in the transplanted side than the control side (21.3 ± 8.1 vs. 11.0 ± 4.6 at 3 minutes, 26.7 ± 6.8 vs. 19.7 ± 5.9 at 5 minutes), while in the sham group, there were no significant differences between sides. In minipigs, lipiodol lymphangiography (n = 5) showed Lipiodol accumulation in transplanted LNs with innumerable newly formed lymphatic vessels and lymphovenous shunts. MR lymphangiography (n = 5) showed higher enhancement on the transplanted side compared to the control side. Histology showed successful engraftment of transplanted LNs in 16 out of 20 (80%) mice and 9 out of 12 (75%) minipigs. Omnidirectional lymphangiogenesis forming a dense lymphatic network and spontaneous formation of lymphovenous shunts were shown after NVLNT.

## Introduction

The lymphatic system, which consists of lymphatic vessels, lymphoid organs, and lymph nodes, is a part of the circulatory system. Lymphatic flow impairment can result from lymphatic tract obstruction, which can cause lymphatic fluid stasis and protein accumulation in the interstitial space and, ultimately, lymphedema [1]. Because the lymphatic system carries antigens and immune effector cells, lymphedema can cause several immunological conditions. Repairing the impaired lymphatic system is important in the treatment of lymphedema [2].

To repair a damaged lymphatic system and promote lymphangiogenesis, lymph node transplantation (LNT) has been suggested for treatment of post-surgical lymphedema [3]. There are two types of LNT: vascularized LNT (VLNT) and non-vascularized LNT (NVLNT) [4]. In VLNT, surrounding lymphatic vessels around a transplanted LN are preserved in a fat flap. In NVLNT, surrounding vessels and fat are removed, and only the LN itself is transplanted.

The process of establishing new lymphatic connections after transplantation is slightly different between VLNT and NVLNT. In VLNT, pre-existing lymphatic vessels in the flap are preserved and are rerouted in the new transplantation location after lymphangiogenesis establishes new connections at the flap border [5]. In NVLNT, however, only the lymph node itself is preserved. All lymphatic vessels are cut at the capsule level. Therefore, rerouting can occur, but only at the capsule level, and lymphangiogenesis is still necessary to establish new connections [6]. Thus, the preservation of the capsule of the lymph node is known to play an important role in the viability of the transplanted LN [7]. However, the establishment of new connections between the transplanted LN and local lymphatic vessels at the transplant site in NVLNT is still poorly understood.

Recently, advanced lymphangiography techniques have opened up new horizons in imaging of the lymphatic system. Ultrasound-guided intranodal lymphangiography directly punctures the LN, and delivers contrast media into the lymph node [8]. It saturates the LN with contrast media thus allowing visualization of the surrounding lymphatic vessel morphology as well as lymphatic flow. The purpose of this study was to investigate lymphatic connections after NVLNT using lymphangiography.

## Materials and methods

The study protocol was approved by the Experimental Ethics Committee of Jeonbuk National University Hospital and Osong Medical Innovation Foundation conformed to the US National Institutes of Health Guidelines on the care and use of laboratory animals. All experiments were performed under international guidelines for animal care and experimental studies.

### Mice model

#### Nonvascularized lymph node transplantation in mice

**Fig 1:**
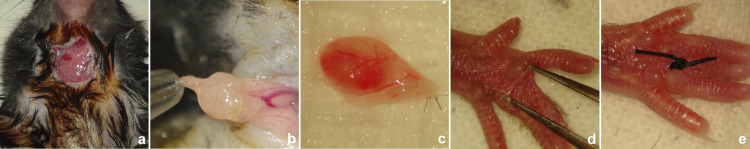
Surgical procedure of NVLNT in mice. (a) Cervical midline incision was made. (b, c) Cervical LN was dissected and harvested. Adjacent vascular structure was carefully cut by cauterization. (d, e) A subcutaneous pocket was made on the plantar side of the left hindlimb. A harvested LN was inserted in the pocket, followed by skin suture.

Male C57/BL6 mice were used for small animal experiments. A total of 40 mice were divided into 2 groups: an NVLNT group and a sham procedure group. All mice were anesthetized by intraperitoneal injection of tiletamine/zolazepam (Zoletil® 50; Virbac, France, 30 mg/kg) and xylazine hydrochloride (Rompun®; Bayer, Germany, 10 mg/kg). All mice were clipped, and residual hair was removed with a depilatory cream in the neck and hindlimb. In the sham procedure group, cervical midline and plantar incisions were performed, but the skin was closed without LN harvest or transplantation. In the NVLNT group, after the sterilization of the cervical and hindlimb skin, a midline incision was done in the cervical area. A cervical LN was carefully dissected and harvested from the cervical fat pad. Connecting vessels were cut by cauterization to minimize vascular injury and bleeding. The harvested LN was placed in a sterile 0.9% saline-soaked gauze. An approximately 2-mm-sized incision was done on the plantar side of the left hind limb. Then, an approximately 3-mm-sized subcutaneous pocket was made with the tip of a fine point forcep. The harvested LN was inserted in the pocket, followed by a skin suture with 5–0 nylon. (Fig 1).

### Indocyanine green fluorescence near-infrared imaging in mice

**Fig 2:**
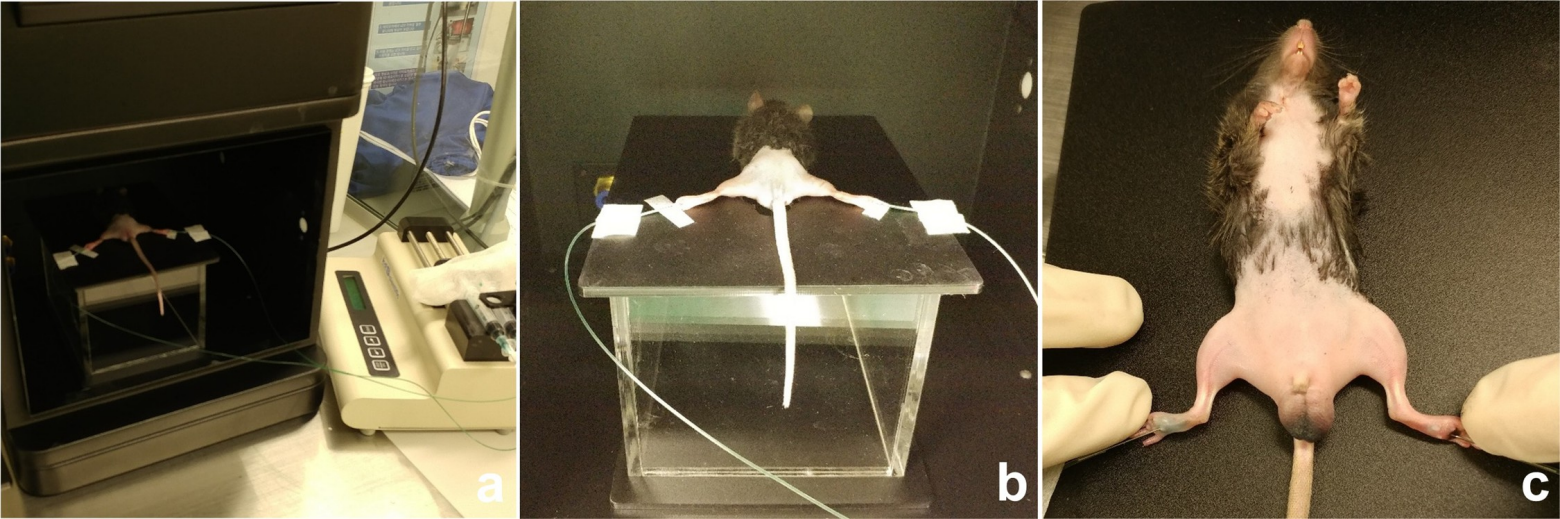
Fluorescent lymphangiography procedure. (a, b) Mouse lying in the chamber. The puncture was made on both sides with 30-gauge needles connected to long tubes and syringes mounted in an automated syringe pump. Dorsal aspect images at baseline, 1, 3, and 5 minutes were obtained. (c) After 5 minutes of continuous injection, the needle and tube were removed. The mouse was turned upside down to take a ventral aspect image.

On day 21 after NVLNT, the lymphatic vessels were observed using a Fluorescence labeled Organism Bioimaging Instrument (FOBI; NeoScience, Korea). Body hair was removed using depilatory cream to avoid autofluorescence from the hair. After anesthesia, an indocyanine green (ICG) solution (1 mg/ml, 1 ul/min, 5 minutes) was injected subcutaneously into the foot pads of both hindlimbs by using an automated syringe pump (KDS 100 legacy syringe pump; KD Scientific, Korea). The near-infrared (NIR) fluorescence images were captured at baseline (before injection) and at 1, 3, and 5 minutes after injection. Image analysis was performed using ImageJ imaging software (U. S. National Institutes of Health, U.S). On each image, the same-sized circular region-of-interest (ROI) was drawn in both popliteal LN and pelvic LN regions. Signal intensities in each ROI were measured, and then signal intensity ratios between the NVLNT and normal sides were calculated in the popliteal and the pelvic regions (Fig 2).

### Histology in mice

After ICG fluorescence NIR imaging, all mice were euthanized using carbon dioxide asphyxiation, and then the left hindlimb of each mouse was collected. The collected hindlimb was immersed in paraformaldehyde perfusion fixative overnight at 4°C. Decalcification was performed, then the samples were embedded in paraffin and sectioned into 5-μm slices. To evaluate the histologic findings, slides were stained with hematoxylin and eosin (Sigma-Aldrich, St Louis, MO, USA). Immunohistochemical staining was done by using an anti-lymphatic endothelial hyaluronan receptor (LYVE-1) polyclonal antibody (Abcam; Cambridge, UK) at a dilution of 1:100. The transplantation was considered successful when the tissue exhibited necrosis of less than 10% and viable cells were observed in 80% or more of the transplanted lymph nodes. If transplanted LN was not identified in histology, LNT was considered as a failure. Data obtained from LNT failures were not used in image analysis.

### Minipig model

#### Nonvascularized lymph node transplantation in minipigs

Twelve male minipigs, weighing 7–13 kg, were used for the large animal experiments. All animals were sedated with intramuscular injection of tiletamine/zolazepam (Zoletil® 50; Virbac, France, 0.5 mg/kg) and xylazine hydrochloride (Rompun®; Bayer, Germany, 1 mg/kg). Each animal was mechanically ventilated for the duration of the experiment with 1% isoflurane inhalation anesthesia (Ifran; Hana Pharma, Korea).

**Fig 3:**
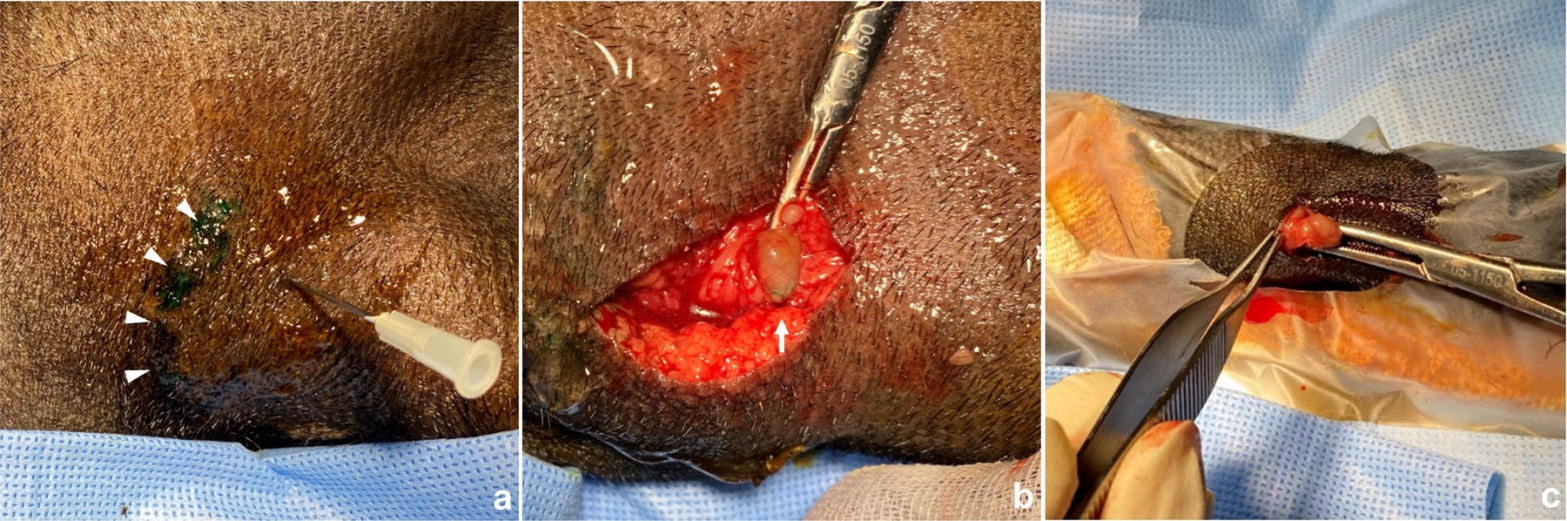
Surgical procedure of NVLNT in minipigs. a, b) The right cervical area was scanned by ultrasound and the location of the cervical LN was marked by an 18-gauge needle. Multiple injections of 0.5 ml of ICG were performed 1–2 cm distal to the needle for staining so the operator could easily identify the cervical LN. c) A subcutaneous pocket was made on the dorsal side of the right hindlimb. The harvested LN was inserted in the pocket, followed by skin suture.

After sterilization of the right cervical area and right hindlimb, the right cervical area was scanned by ultrasound. A prominent cervical LN was identified by ultrasound and marked by an 18-gauge needle. An intradermal injection of 0.5 ml of diluted indocyanine green (Indocyanine Green inj; Dongindang, Korea, 5mg/ml) was performed 4 to 5 times 1–2 cm distal to the needle marking site. A 2–3 cm sized vertical skin incision was performed, and subcutaneous fat separation was done to localize the stained cervical LN. The LN was carefully dissected and harvested from the cervical fat, then immersed in normal saline. An approximately 1-cm-sized transverse incision was done on the dorsal side of the right hindlimb. Then, an approximately 2-cm-sized subcutaneous pocket was made with the tip of a forcep. The harvested LN was inserted into the pocket. Skin incision sites were closed with suture clips (Fig 3).

### Lipiodol lymphangiography and magnetic resonance lymphangiography in minipigs

On day 14 after NVLNT, the transplanted lymph node and donor site were checked by ultrasound. If there was no identifiable LN in the transplanted site, the minipig was excluded from lymphangiography. The remaining minipigs were divided into 2 groups. One group was assigned to lipiodol (Guerbet, France) lymphangiography, and another group was assigned to magnetic resonance (MR) lymphangiography with gadobenate dimeglumine (Gd-BOPTA, Multihance, Bracco Imaging, Italy). Before the lymphangiography, general anesthesia was performed in the manner described above. Needle insertion and fixation were performed in both groups in the same manner. With the minipig in the supine position, an ultrasound-guided puncture in the transplanted LN was performed using a 26-gauge spinal needle (Hakko, Japan). Another spinal needle was inserted in the dorsum of the opposite foot. Extension tubes were connected to both needles. On the LNT side, a trace amount of lipiodol was injected to confirm the needle was well located in the LN. After confirming the needle was located in the LN, the needle was fixed to the skin by applying a bandage.

In the lipiodol lymphangiography group, lipiodol was injected in both sides at a rate of 1 ml/5 min. The flow of lipiodol was monitored by real-time fluoroscopy until both inguinal LNs were opacified. In the MR lymphangiography group, all MR examinations were performed on a preclinical 4.7 T MR imaging device (Biospec 47/40, Bruker BioSpin, Germany). For MR lymphangiography, a 3D T1-weighted gradient-echo sequence (FLASH) was used. The MR imaging parameters were as follows: TR/TE 30/3.1, flip angle 25, FOV 200X200X80 mm3, matrix 256X256, slice thickness 2.5 mm, acquisition time 3 minutes. MR images were obtained before the injection of Gd-BOPTA. Then, 2 ml of Gd-BOPTA was manually injected in both sides at once. MR lymphangiography images were obtained every 8 minutes for 32 minutes following injection.

### Histology in minipigs

After lymphangiography, all minipigs were euthanized. The right hindlimb of each minipig was collected. The collected hindlimb was immersed in paraformaldehyde perfusion fixative overnight at 4°C. The skin, soft tissue, and transplanted LN were removed; then the samples were embedded in paraffin and sectioned into 5-μm slices. The slides were stained with hematoxylin and eosin. Immunohistochemical staining was done by using an anti-lymphatic endothelial hyaluronan receptor (LYVE-1) polyclonal antibody (Abcam; Cambridge, UK) at a dilution of 1:100. The transplantation was considered successful when the tissue exhibited necrosis of less than 10% and viable cells were observed in 80% or more of the transplanted lymph nodes.

## Results

### Mice

**Fig 4:**
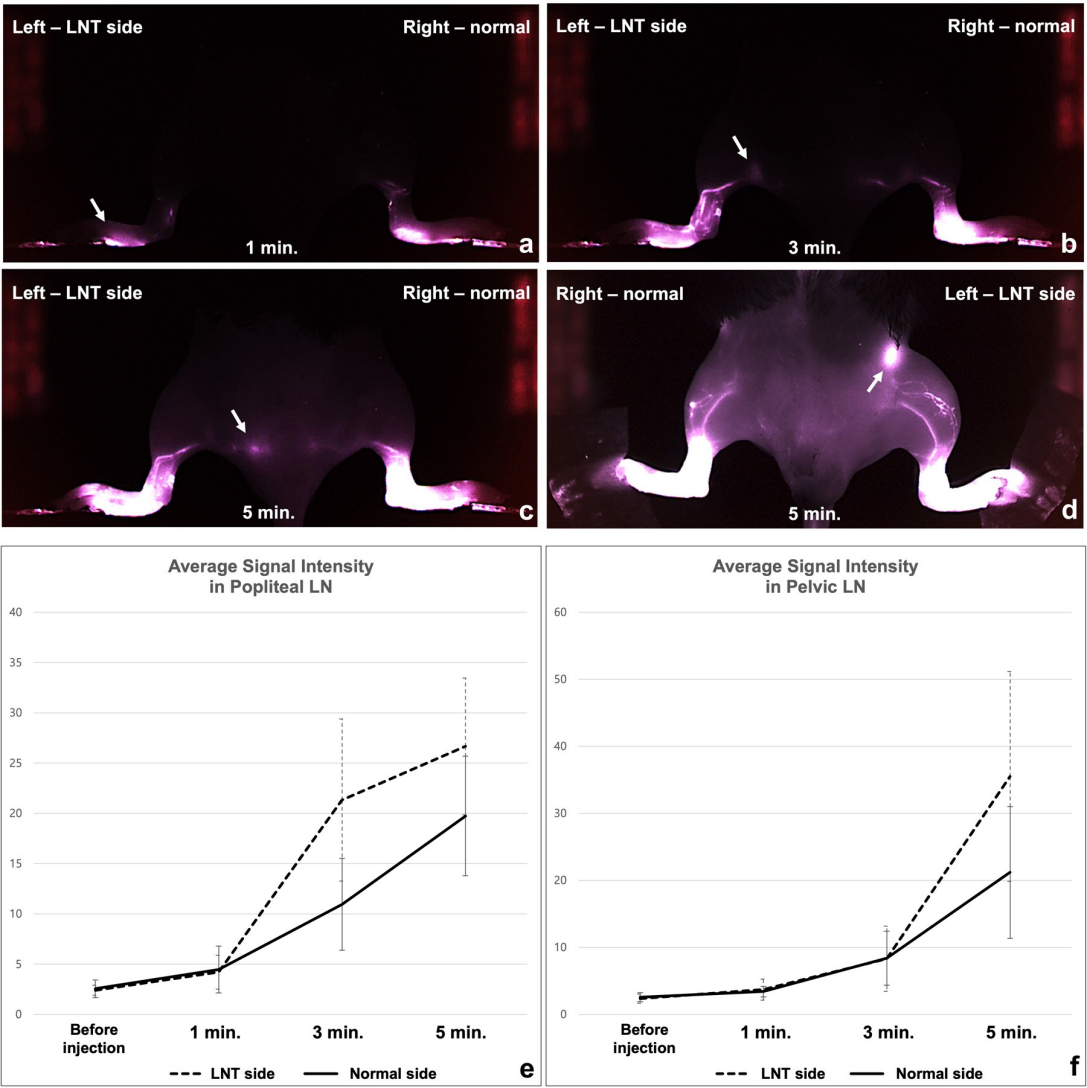
Fluorescent images with continuous ICG injection in both fat pads. (a) Dorsal aspect image at 1 minute after starting injection. There were no significant differences between the LNT and normal sides. Arrow indicates the LNT site. (b) Dorsal aspect image at 3 minutes after starting injection. ICG accumulation in the popliteal LN is more evident on the LNT side compared to the normal side (arrow). (c) Dorsal aspect image at 5 minutes after starting injection. ICG accumulation in the pelvic LN is more evident on the LNT side compared to the normal side (arrow). (d) Ventral aspect image at 5 minutes after starting injection. ICG accumulation in the inguinal LN is well observed. (e, f) Graph shows higher signal intensities in the popliteal LN of the LNT side at 3 minutes and 5 minutes compared to the normal side, while a higher signal intensity in the pelvic LN was observed only at 5 minutes.

All ICG NIR examinations were successfully performed, but only mice with histologically-proven successful LNT were included in the final image analysis. ICG administered to the hind paw was drained to the popliteal lymph node, then to the ischial lymph node of the pelvic region in all mice. Fig 4 shows fluorescent images of the dorsal and ventral aspects in mice with LNT. In the popliteal region, the signal intensities at 3 minutes and 5 minutes were higher in the LNT side than the control side (21.3 ± 8.1 vs. 11.0 ± 4.6 at 3 minutes, 26.7 ± 6.8 vs. 19.7 ± 5.9 at 5 minutes). Signal intensities at 1 minute were not significantly different between the LNT and control sides (4.2 ± 1.7 vs. 4.5 ± 2.3). In the pelvic region, only the signal intensity at 5 minutes was higher in the LNT side than the control side (35.5 ± 15.6 vs. 21.2 ± 9.8 at 5 minutes). The signal intensities at 1 minute and 3 minutes were not significantly different between the LNT and control sides (3.7 ± 1.6 vs. 3.4 ± 0.8 and 8.3 ± 4.9 vs. 8.4 ± 4.0, respectively). In the sham procedure group, there were no significant differences in popliteal and pelvic signal intensity at any time points between two hind limbs.

**Fig 5:**
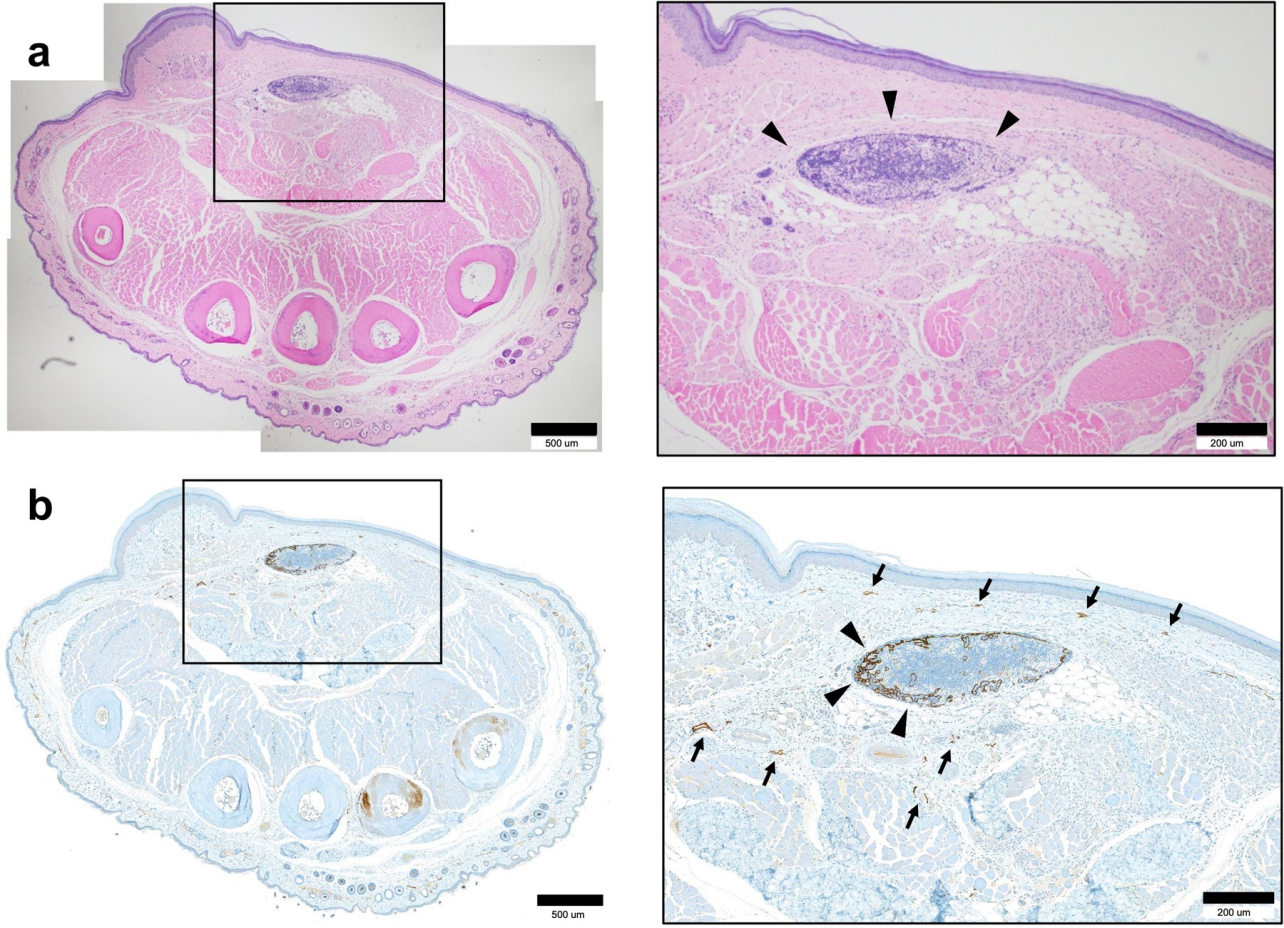
Histology of a transplanted LN in mice. (a) The transplanted LN (arrowheads) was observed in the foot pad on H&E staining. Scale bar left 500 um, right 200 um. (b) The transplanted LN was stained with an anti-LYVE-1 antibody. LYVE-1 is expressed on the surface of the transplanted LN (arrowheads). A number of LYVE-1 stained lymphatic vessels were observed around the LN (arrows).

Successful engraftment of the transplanted LN was found in 16 of 20 mice (80%) on histology (Fig 5). In mice with successful engraftment, the transplanted LN was found in the paw with a thick outer layer of LYVE-1. Multiple scattered lymphatic vessels were found around the LN. In mice with failed engraftment, no viable lymph node or necrotic foci were found.

### Minipigs

Ten of 12 transplanted lymph nodes were identified on US examination at 2 weeks. Two showed fluid collection without a visible lymph node in the transplanted site, suggesting abscess formation. In donor sites, 1 showed fluid collection suggesting a lymphocele. Ten minipigs with a remaining transplanted lymph node were divided into 2 groups for Lipiodol (5 minipigs) and MR lymphangiography (5 minipigs).

**Fig 6:**
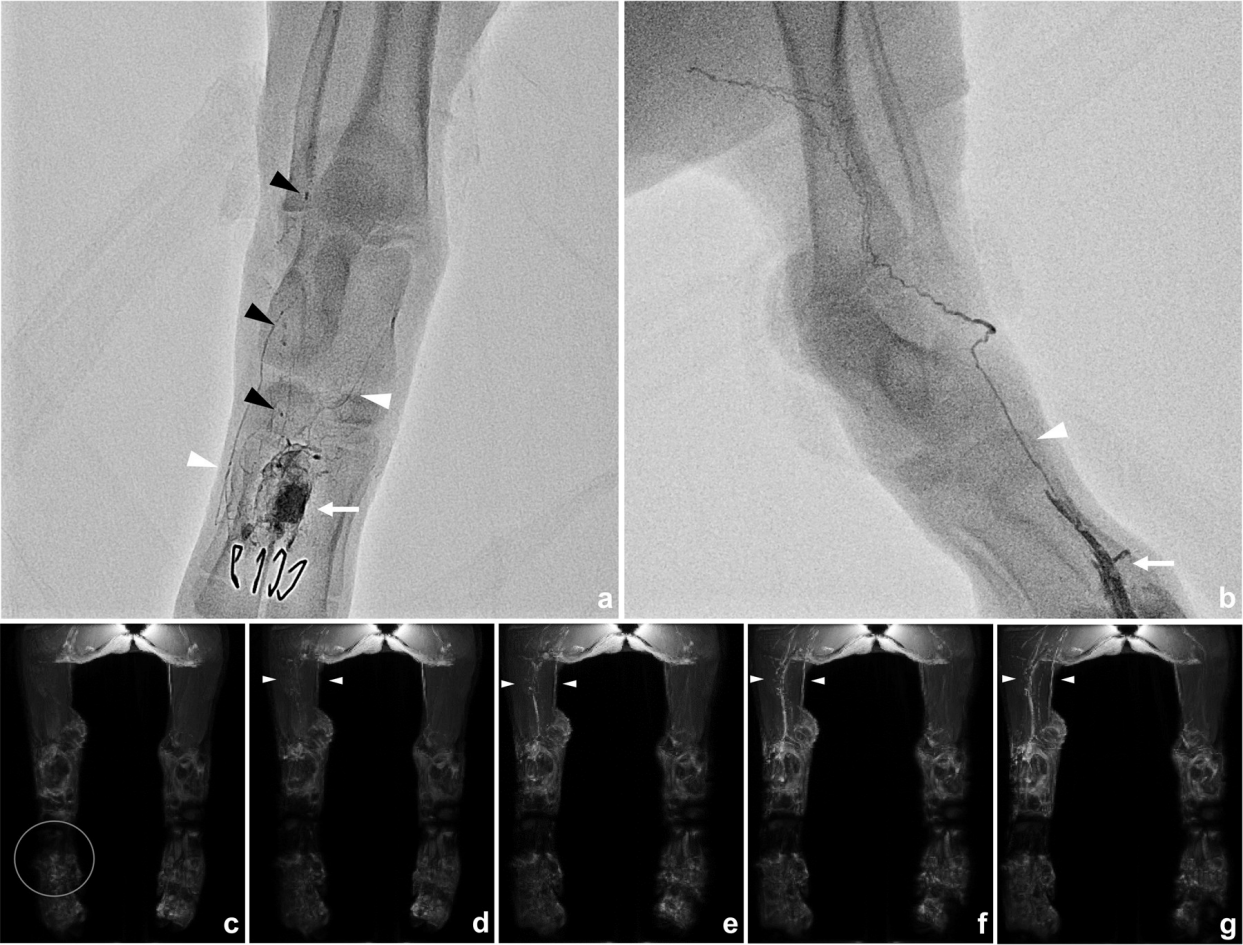
Lipiodol and MR lymphangiography in minipigs. (a) Lipiodol lymphangiography by intranodal lymphangiography shows the transplanted LN and a dense lymphatic network around the LN in the dorsum of the foot (arrow). Lipiodol fills in adjacent lymphatic vessels (white arrowheads), and the oil-in-water state of lipiodol flow through the lymphovenous shunt is observed as a string of beads (black arrowheads). (b) On the normal side, extravasation of lipiodol is observed (arrow). The injected lipiodol fills a single lymphatic vessel without a distinct lymphatic network or lymphovenous shunt. (c-g) Dynamic MR lymphangiography by intranodal injection of Gd-BOPTA. (c) Before injection, (d) 8 minutes delay, (e) 16 minutes delay, (f) 24 minutes delay, and (g) 32 minutes delay. Two ml of Gd-BOPTA was injected into a transplanted LN (circle) and the dorsum of the normal hindlimb. Faster filling of lymphatic vessels is observed on the LNT side compared to the normal side (arrows). Differences between both sides become more obvious over time.

Lipiodol lymphangiography and MR lymphangiography were successfully performed on all minipigs (Fig 6). Lymphangiography on the LNT side showed Lipiodol accumulation in transplanted LN with innumerable lymphatic vessels formation around the LN. Lymphatic vessels formed a network, and the Lipiodol drained to lymphatic collecting vessels in the hindlimb. A few lymphovenous shunts were observed in all minipigs, which showed rapid and continuous Lipiodol draining during injection. Lipiodol lymphangiography on the control side showed a few lymphatic vessels, which drained to lymphatic collecting vessels in the pelvis.

MR lymphangiography showed higher enhancement in lymphatic collecting vessels of the thigh on the transplanted side compared to the control side. The difference was noticeable over time during MR lymphangiography.

**Fig 7:**
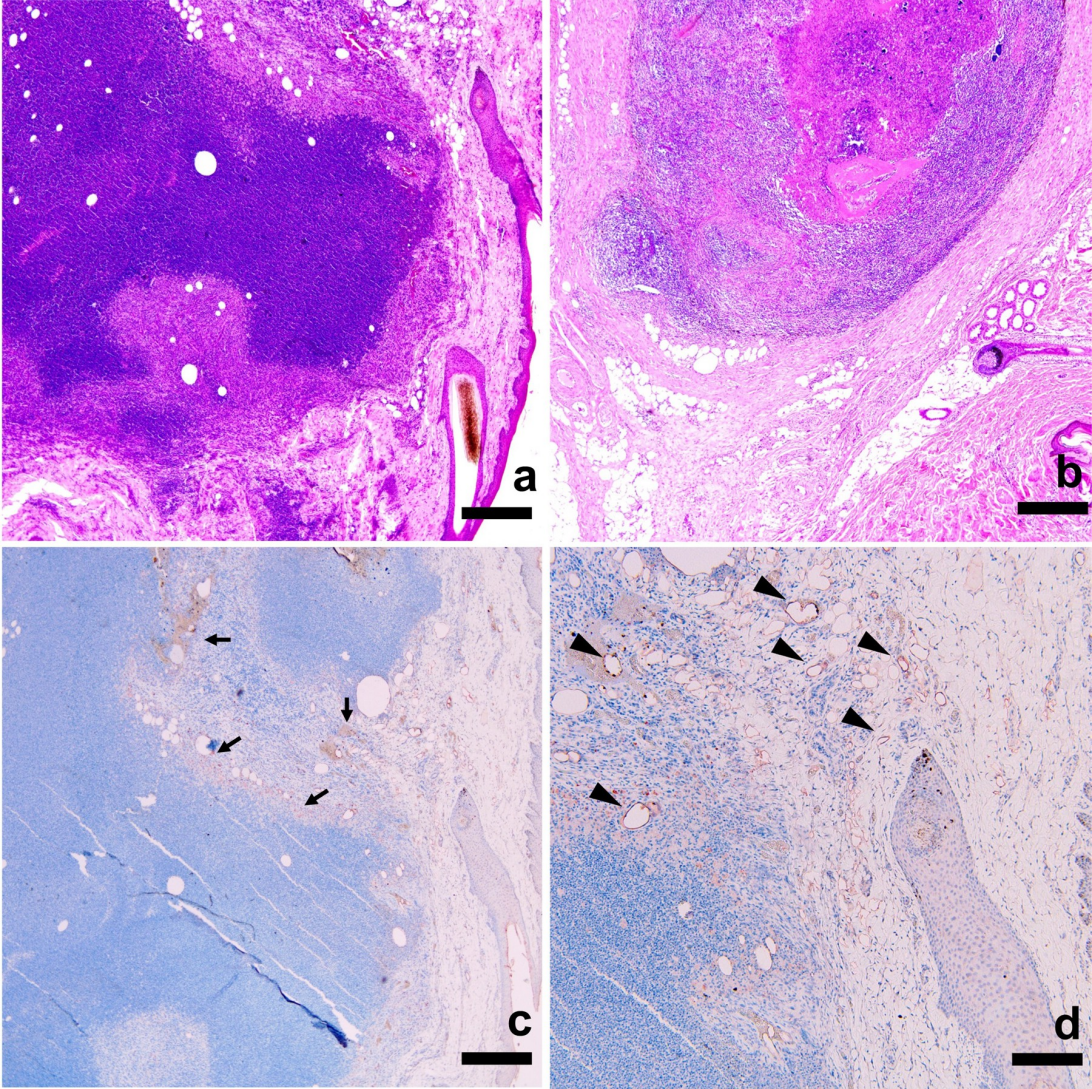
Histology of a transplanted LN in minipigs underwent lipiodol lymphangiography. (a) Numerous viable lymphocytes were observed inside of the transplanted LN suggesting successful engraftment. Scale bar, 200 um. (b) Relatively sparse cellularity with central necrosis was observed inside the transplanted LN suggesting failed engraftment. (c, d) Immunohistochemistry staining of the transplanted LN. A band of staining was observed along the hilum (arrows). Innumerable LYVE-1-stained lymphatic vessels are observed around the transplanted LN. Scale bars, 200 um.

Successful engraftment of the transplanted LN was seen in 9 of 12 minipigs (75%) on histology (Fig 7). In minipigs with successful engraftment, the transplanted LN was found in the dorsum of the foot with innumerable lymphatic vessels around the cortex of the LN.

## Discussion

The main findings in this animal model study were that (1) NVLNT to remote sites was successfully demonstrated with lymphangiography in most cases, (2) NVLNT resulted in omnidirectional lymphangiogenesis between the transplanted LN and local lymphatic vessels, and (3) de novo formation of lymphovenous shunts occurred at the transplanted site.

To investigate lymphangiogenesis after NVLNT in this study, a transplanted LN was harvested from the cervical area and transplanted to the foot where LNs are naturally absent. In a previous study, Maeda et al., demonstrated rerouting of lymphatic vessels after NVLNT [9]. However, in their study, a popliteal LN of a mouse was harvested and transplanted immediately to the same site. Thus, rerouting mainly occurred between the transplanted LN and the popliteal lymphatic collecting duct which was originally connected to the popliteal LN. However, LNT to the same site is not feasible from a clinical perspective. The engraftment of a transplanted LN and lymphangiogenesis might be more difficult after NVNLT to a remote site because there are structural differences as well as a sparse lymphatic vessel density compared to the donor site. Thus, we believe our model might better reflect practical scenarios of NVLNT, and might represent the genuine capability of lymphangiogenesis after NVLNT.

The 2 main mechanisms of VLNT are lymphangiogenesis, with new lymphatic collateral pathways connected to adjacent lymph nodes and mediated by lymphatic growth factor secretion from the transplanted LN, and new lymphovenous drainage formation within the transplanted LN [10–13]. We found similar processes occurring after NVLNT in this study. Interstitial flow is known to facilitate lymphangiogenesis in specific directions by guiding lymphatic cellular migration [14]. In this study, a nonvascularized LN was transplanted to the foot pad or dorsum where interstitial flow is limited, leading to omnidirectional lymphangiogenesis with a dense lymphatic network around the transplanted LN. This indicates that a nonvascularized LN acted as a center of lymphangiogenesis without the surrounding fat flap; thus, it might be used as the sole treatment option for lymphedema or as an adjuvant method that supports vascularized LN transplantation.

Anastomosing lymphatic vessels to adjacent veins redirect lymphatic flow to the venous circulation, thus evacuating = excess lymphatic fluid to the venous circulation. This demonstrates a considerable treatment effect in secondary lymphedema with persistent effectiveness [15]. However, the main limitations of the microsurgical procedure include limited availability, technical difficulty, and the need for dedicated logistics and expertise. Therefore, this treatment could be only considered in dedicated centers. The spontaneous development of a lymphovenous shunt within the transplanted LN could act as a similar therapeutic mechanism in VLNT [13]. In this study, we found the formation of several lymphovenous shunts after NVLNT. Angiogenesis accompanied by a lymphovenous shunt seems to have spontaneously occurred following lymphangiogenesis due to the regeneration of disconnected blood vessels in the hilum of the non-vascularized LN. Taken together, NVLNT may establish a lymphatic network and a lymphovenous shunt at the transplant site similar to VLNT, which may have therapeutic potential in secondary lymphedema.

This study also revealed that intranodal lymphangiography is a feasible technique in a transplanted LN. Intranodal lymphangiography is a state-of-art technique currently being used in thoracic duct imaging [8, 16, 17]. Micro-sized lymphatic vessels could be visualized grossly in real-time under high-resolution fluoroscopy in our study, enabling visualization of a lymphovenous shunt. Also, contrast media could be effectively absorbed in the lymphatic system with minimal extravasation in the injection site, leading to fast lymphatic imaging. To our knowledge, there is no previous study about the application of the intranodal lymphangiography technique in transplanted LNs. We have demonstrated that intranodal lymphangiography is a feasible technique in transplanted LNs. The drainage function of a transplanted LN could be evaluated by this method; thus, this technique could be used in the postoperative evaluation of LNT.

Future study is warranted to elucidate unclear issues regarding the pattern of lymphangiogenesis. In this study, LN transplantation was not performed under pathologic conditions such as lymphedema. In most clinical situations, the target site of LNT has extensive fibrosis due to previous surgery and irradiation; thus, the potential of lymphangiogenesis may be limited. It is uncertain whether lymphangiogenesis and lymphovenous shunts could progress in these unfavorable environments. Another issue is whether, in addition to the connection of lymphatic vessels, lymph node function is preserved after NVLNT. One of the most important functions of an LN is to present tumor-derived antigens to naive T cells [18]. Although Maeda et al. showed preservation of the T cell and B cell population after the NVLNT in their study [9], further research is needed to clarify whether the same occurs in NVNLT in remote sites. Furthermore, it would be beneficial to conduct a comparison study with VLNT. This would help to elucidate the differences in viability and patterns of lymphangiogenesis between the two methods, and to determine which approach may be more advantageous in specific clinical scenarios.

## Conclusions

In conclusion, we have demonstrated omnidirectional lymphangiogenesis after NVLNT. Regenerated lymphatic vessels formed dense lymphatic networks with surrounding lymphatic vessels, and lymphatic flow through the network was established. Furthermore, spontaneous formation of lymphovenous shunts occurred after NVLNT.
